# The diagnostic and prognostic value of radiomics and deep learning technologies for patients with solid pulmonary nodules in chest CT images

**DOI:** 10.1186/s12885-022-10224-z

**Published:** 2022-11-01

**Authors:** Rui Zhang, Ying Wei, Feng Shi, Jing Ren, Qing Zhou, Weimin Li, Bojiang Chen

**Affiliations:** 1 Department of Pulmonary and Critical Care Medicine, West China Hospital, Sichuan University, 37 GuoXue Alley, Wuhou District, Chengdu, Sichuan Province 610041 People’s Republic of China; 2 Department of Research and Development, Shanghai United Imaging Intelligence Co., Ltd., Shanghai, China

**Keywords:** Solid pulmonary nodules, Radiomics, Deep learning, Adenocarcinoma, Disease-free survival

## Abstract

**Background:**

Solid pulmonary nodules are different from subsolid nodules and the diagnosis is much more challenging. We intended to evaluate the diagnostic and prognostic value of radiomics and deep learning technologies for solid pulmonary nodules.

**Methods:**

Retrospectively enroll patients with pathologically-confirmed solid pulmonary nodules and collect clinical data. Obtain pre-treatment high-resolution thoracic CT and manually delineate the nodule in 3D. Then, all patients were randomly divided into training and testing sets at a ratio of 7:3, and convolutional neural networks (CNN) models and random forest (RF) models were established. Survival analyses were performed for patients with solid adenocarcinomas.

**Results:**

Totally 720 solid pulmonary nodules were enrolled, 348 benign and 372 malignant. The CNN model with clinical features achieved the highest AUC [0.819, 95% confidence interval (CI): 0.760–0.877] with a sensitivity of 0.778, specificity of 0.788 and accuracy of 0.783. No significant differences were observed between the CNN and radiomics models. There were 295 solid adenocarcinomas in survival analysis. Different disease-free survival was observed between the low-risk and high-risk groups divided according to the radiomics Rad-score. However, the groups based on deep learning signatures showed similar survival. Cox regression analysis indicated that the radiomics Rad-score (hazard ratio: 5.08, 95% CI: 2.61–9.90) was an independent predictor of recurrence.

**Conclusions:**

The radiomics and deep learning models can well predict the malignancy of solid pulmonary nodules. Radiomics signatures also demonstrate prognostic value in solid adenocarcinomas.

**Supplementary Information:**

The online version contains supplementary material available at 10.1186/s12885-022-10224-z.

## Background

Lung cancer screening with low-dose computed tomography among high-risk individuals can reduce lung cancer modality [1, 2]. However, it’s challenging to manage pulmonary nodules detected on thoracic CT either during screening or routine clinical practice. Solid pulmonary nodules are usually distinct from subsolid nodules (SSNs) and therefore different recommendations were provided in guidelines [3–6]. Most SSNs exhibit indolent nature and grow slowly or stay stable over years [7], and often pathologically diagnosed as lung adenocarcinomas [8]. However, the solid nodules which could be caused by various respiratory diseases, can grow rapidly and are more prone to distant metastasis when they are malignant [9, 10]. Therefore, the risk prediction of solid pulmonary nodules should be important, as it can help clinicians make the right decision and save time for patients during medical care.

Recently, radiographic assessment of disease is being improved by advanced computational analyses. On the one hand, radiomics approach can digitally decode radiographic images into quantitative features (e.g., descriptors of shape, size and textural patterns), and therefore classify the medical image into a predefined group [11]. On the other hand, deep learning has made great strides in automatically characterizing radiographic images. It uses convolutional neural networks (CNN) to automatically learn feature representations from sample images, which could match and even surpass human performance in task-specific applications [12].

In previous studies, researchers have investigated the diagnostic performance of radiomics and deep learning technologies for solid pulmonary nodules [13–19]. However, some of the studies focused mainly on small solid pulmonary nodules, such as nodules less than 15 mm or 20 mm [14, 15, 17]. Some studies differentiated solid nodules between one specific benign lung disease and lung adenocarcinoma, like focal organizing pneumonia, solitary granulomatous nodules or tuberculosis [13, 16, 19]. Besides, none of the studies investigated prognostic values of radiomics and deep learning technologies for solid nodules.

Therefore, the current study intended to establish CNN and radiomics models for solid pulmonary nodules without restricting the nodule size and pathology. Furthermore, survival analyses were performed for patients with solid adenocarcinomas.

## Methods

### Patients and clinical variables

This retrospective study was approved by the institutional review board of the West China Hospital of Sichuan University. We collected possible cases by reviewing discharge records of patients in West China Hospital from January 2010 to July 2017. The following terms were used to extract the data: lung cancer, lung adenocarcinoma, lung squamous carcinoma, non-small cell lung cancer, small cell lung cancer; inflammatory lung nodule, benign lung nodule, benign lung tumor, lung hamartoma, lung sclerosing hemangioma, lung tuberculosis, lung granuloma. Then, the patient was enrolled based on the following criteria: (a) there was an untreated, pathologically confirmed, 5–30 mm noncalcified solid nodule detected on chest CT; (b) the slice thickness of CT was less than or equal to 1 mm. Otherwise, patients were excluded if (a) there were multiple pulmonary nodules, or pleural effusion, atelectasis, lymph node enlargement was observed; (b) it wasn’t a primary lung tumor.

Totally, the current study enrolled 720 patients with 720 nodules, 348 benign and 372 malignant. The pathology of benign nodules was confirmed by surgery (*N* = 315, 90.5%) and CT guided percutaneous lung biopsy (*N* = 33, 9.5%), while the malignant nodules was confirmed by surgery (*N* = 365, 98.1%), CT guided percutaneous lung biopsy (*N* = 4, 1.1%) and transbronchial lung biopsy (*N* = 3, 0.8%), respectively.

Following clinical characteristics were recorded, including age, sex, smoking status, history of malignancy, family history of malignancy, nodule diameter, location, pathology and clinical stage. As surgically resected adenocarcinomas were predominant among all malignant nodules, prognostic data were collected for survival analysis.

### CT image acquisition and nodule segmentation

Thoracic CT before treatment was obtained for each patient. All images were acquired from GE, Siemens or Philips scanners, with tube voltage and current being 100 ~ 120 Kvp and 60 ~ 250 mAs. Reconstructions were performed using a standard convolution kernel. The detailed information on manufacturer, manufacturer’s model and slice thickness were summarized in Table S1 and Table S2.

All target nodules were first manually segmented in 3D by one author with 4 years of clinical experience in pulmonology, using the ITK-SNAP software. Then, randomly selecting 100 patients, both the same author and another author manually segmented the target nodules again to assess the consistency of the intra-rater and inter-rater segmentations by calculating Dice similarity coefficient. Both authors were blinded to pathological results of lesions.

### CNN models

**Fig. 1:**
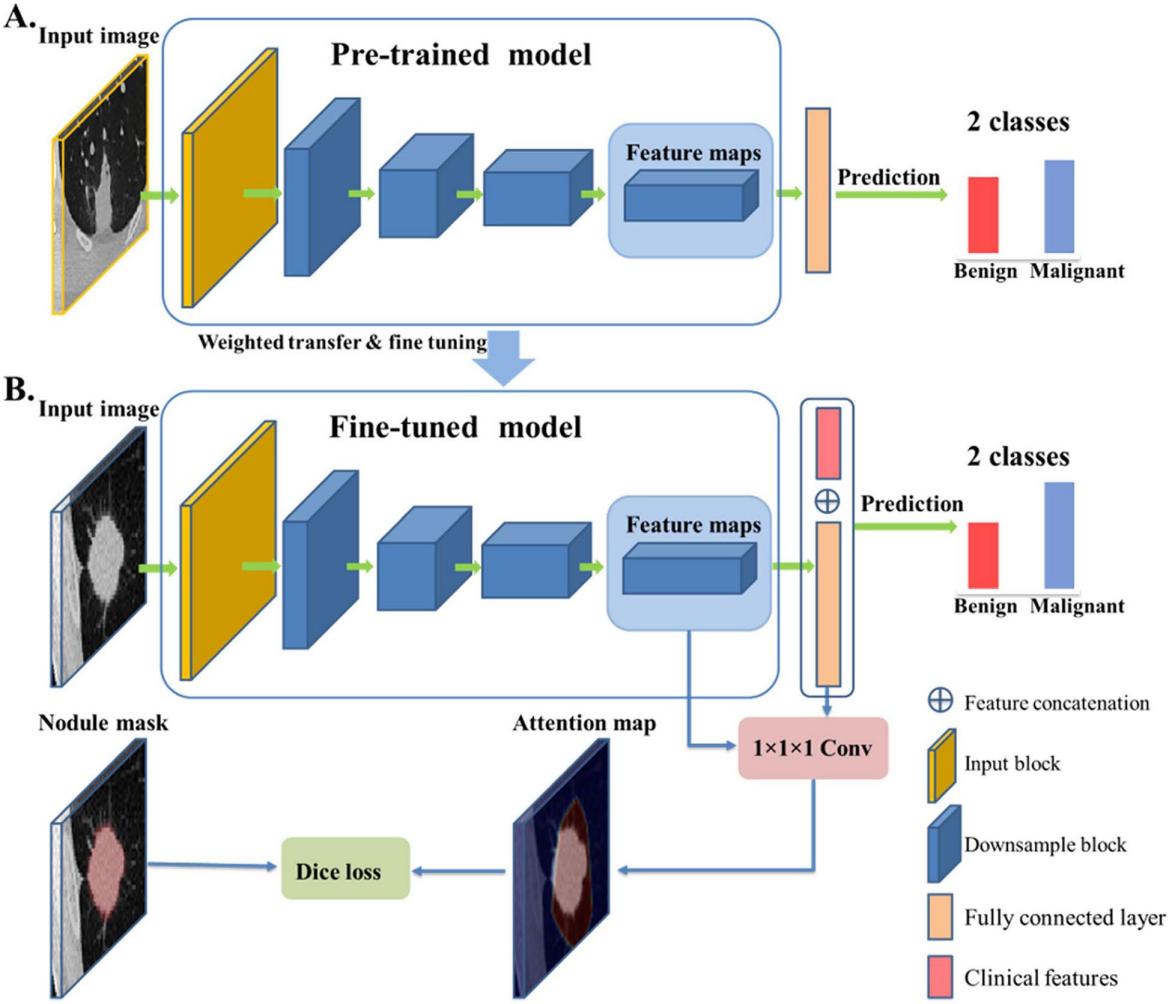
The framework of the deep learning model. **A** The pre-trained model. **B** The proposed model. “1 × 1 × 1 Conv” refers to the convolutional layer with 1 × 1 × 1 kernel. Dice loss refers to Dice similarity coefficient between the nodule mask and class activation mapping (attention map)

Patients were randomly divided into training and testing set at a ratio of 7:3 during model establishment. The overall framework of the CNN model is shown in Fig. 1. Here we used transfer learning from a pre-trained benign-malignant nodule classification model, in which 1715 pathologically-confirmed nodules and 14,735 unlabeled nodules were used [20]. In detail, there were one 3D convolution layer with a kernel size of 3 and stride of 1 as input block, four 3D convolution layers with a kernel size of 3 and stride of 2 as downsample block, and two fully connected layers as output block for the benign-malignant classification task. Besides, the class activation mapping was used to guide the network focusing on the nodule region, where attention maps were generated by back-propagating weights of the fully-connected layer onto the convolutional feature maps [21]. In total, two CNN models were established based on whether clinical features were added.

### Radiomics models

Firstly, radiomics features were extracted of segmented nodules, including 42 dedicated handcrafted features and 104 widely-used radiomics features. Details of handcrafted features were described in previous study [22]. The widely-used first-order image intensity statistics, shape and texture features were extracted using PyRadiomics [23]. Then, three RF models were established by using radiomics features, clinical features and both features, respectively. To avoid overfitting and obtain predictive features, least absolute shrinkage and selection operator (LASSO) was applied for radiomics feature selection where regression coefficients of irrelevant variables were shrunk to zero. To achieve the best performance, an optimization algorithm based on Bayesian was used to optimize the hyperparameters.

To compare the diagnostic performance of established models with manual visual assessment, two junior radiologists were invited to blindly classify the solid nodules in the testing set.

### Statistical analysis

The continuous variables, age and nodule diameter, were presented with mean ± standard deviation and compared with Student’s t-test. The follow-up time was compared with Mann-Whitney U test. The other categorical data were described in number of cases (proportion) and compared with Chi-square test.

The classification performance of the models was evaluated on sensitivity, specificity, accuracy, receiver operating characteristic curves (ROC) and values of area under the ROC curve (AUC). Calibration curves were also plotted to evaluate the accuracy of risk estimate. Additionally, Brier scores were calculated that quantitatively measure the distance in the probability domain and a lower score means better prediction. Differences in the AUC values were assessed by Delong test [24].

For prognostic analysis, a Rad-score was computed for each patient by combining LASSO selected radiomics features. According to the Rad-score, patients were classified into low-risk or high-risk group split by X-tile (version 3.6.1, http://tissuearray.org/) [25]. The potential association of radiomics signature with disease-free survival (DFS) was evaluated by Kaplan-Meier survival analysis and multivariate Cox regression. Similarly, the prognostic value of malignancy-score derived from CNN model (with clinical features) was also evaluated. Differences in survival curves were assessed by log-rank test.

The LASSO analysis, ROC curves, calibration curves and Brier scores were implemented with an open source “Scikit-learn 1.1.2” in Python. The Kaplan-Meier survival analysis and multivariate Cox regression were performed with “survival 3.1-8, survminer 0.4.8” packages in R. The statistical tests were all two-sided and differences with *P* < 0.05 were considered statistically significant. All statistical analyses were conducted using R version 3.6.0 and Python version 3.7.0.

## Results

### Patient characteristics

Table 1 describes the clinical characteristics of the enrolled 720 patients. Malignant nodules were mainly lung adenocarcinomas (*N* = 334, 90%) and most belonged to stage I (*N* = 339, 91%). As for benign nodules, they were chronic inflammation (*N* = 112, 32.2%), benign tumor (*N* = 90, 25.9%), tuberculosis (*N* = 75, 21.5%), granuloma (*N* = 67, 19.3%) and so on.

**Table 1:** Clinical characteristics of enrolled patients

Characteristics	Pathology		*P*	Dataset		*P*
	Benign (***N*** = 348)	Malignant (***N*** = 372)		Training (***N*** = 517)	Testing (***N*** = 203)	
Age, year	51 ± 13	60 ± 10	< 0.001*	56 ± 12	55 ± 12	0.058
Sex, male	187 (53.7)	177 (47.6)	0.099	265 (51.3)	99 (48.8)	0.548
Smoking, current or ever smoker	120 (34.5)	142 (38.2)	0.304	192 (37.1)	70 (34.5)	0.505
History of malignancy, yes	11 (3.2)	30 (8.1)	0.005*	33 (6.4)	8 (3.9)	0.203
Family history of malignancy, yes	48 (13.8)	57 (15.3)	0.561	77 (14.9)	28 (13.8)	0.707
Diameter of nodules, mm	17.6 ± 6.1	19.2 ± 5.6	< 0.001*	18.4 ± 5.9	18.4 ± 6.0	0.949
Location of nodules			0.553			0.358
Upper Right	98 (28.2)	118 (31.7)		158 (30.6)	58 (28.6)	
Middle Right	33 (9.5)	34 (9.1)		54 (10.4)	13 (6.4)	
Lower Right	78 (22.4)	69 (18.5)		106 (20.5)	41 (20.2)	
Upper Left	64 (18.4)	78 (21.0)		99 (19.1)	43 (21.2)	
Lower Left	75 (21.6)	73 (19.6)		100 (19.3)	48 (23.6)	

* *P* < 0.05

Unless specified, data in parentheses are percentages

Regarding benign and malignant nodules, the two groups were different in the distribution of patient age, history of malignancy and nodule diameter. Patients with malignant nodules were older (51 ± 13 vs 60 ± 10 years, *P* < 0.001) and exhibited a higher rate of history of malignancy (3.2% vs 8.1%, *P* = 0.005). Besides, malignant nodules tended to be larger than the benign ones (17.6 ± 6.1 vs 19.2 ± 5.6 mm, *P* < 0.001). No significant difference was observed between the two groups regarding to sex, smoking, family history of malignancy and nodule location. There were 244 (47.2%) benign and 273 (52.8%) malignant nodules in the training group, and 104 (51.2%) benign and 99 (48.8%) malignant nodules in the testing group, respectively (*P* = 0.329). No significant difference was observed between the training and testing group.

The Dice similarity coefficient of between-rater and within-rater segmentation was 92.7% and 98.6% respectively, which indicated that the masks had a relatively good consistency.

### Predictive performance of models

**Fig. 2:**
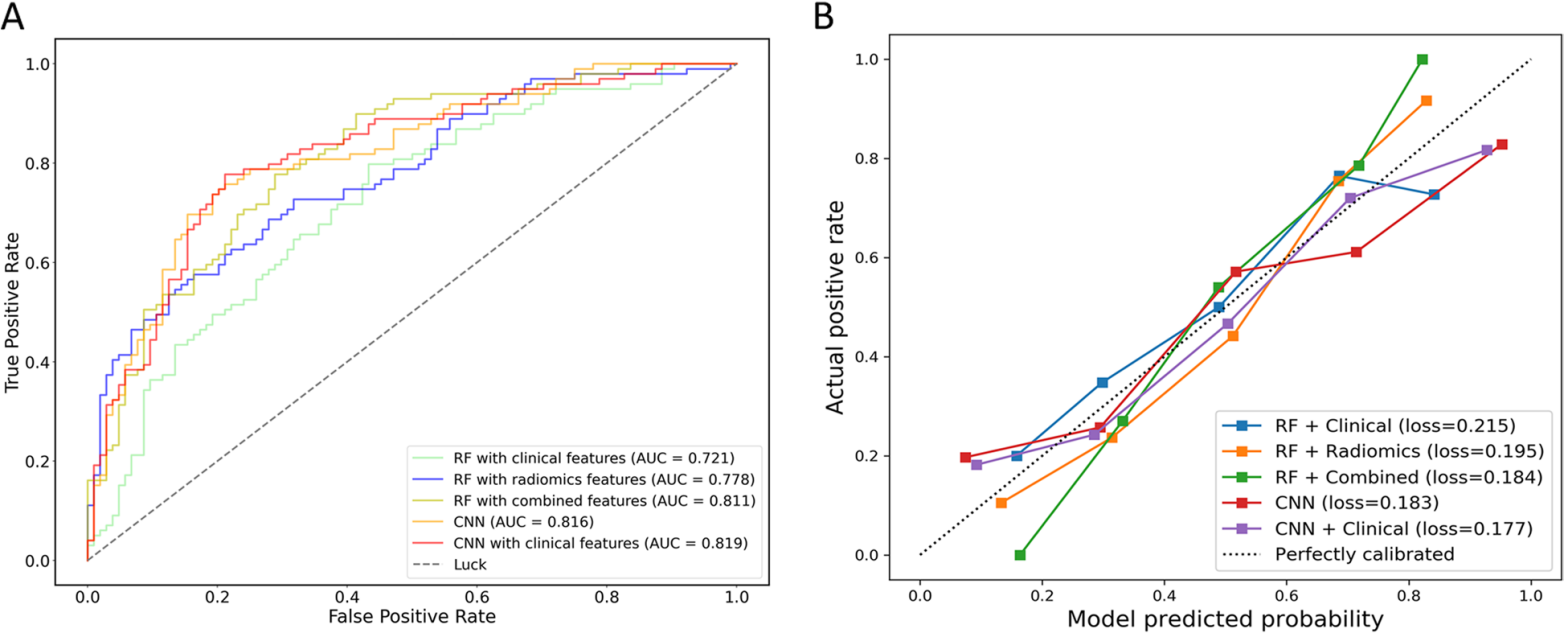
ROC curves (**A**) and calibration curves (**B**) of different classification models in the testing set. RF: random forest, CNN: convolutional neural network

Figure 2A demonstrates the ROC curves of each model in the testing set. With malignant nodules as positive, the CNN model with clinical features achieved the highest AUC [0.819, 95% confidence interval (CI) 0.760–0.877] with sensitivity of 0.778, specificity of 0.788 and accuracy of 0.783. The CNN model without clinical features achieved an AUC of 0.816 (95% CI 0.758–0.875), sensitivity of 0.758, specificity of 0.788 and accuracy of 0.773. In the RF classifier models, the performance of RF with combined features achieved sensitivity of 0.616, specificity of 0.788, and accuracy of 0.704 and AUC of 0.811. In addition, the sensitivity, specificity, accuracy and AUC of RF with radiomics features was 0.747, 0.606, 0.675 and 0.778, respectively. The sensitivity, specificity, accuracy and AUC of RF with clinical features was 0.535, 0.740, 0.640 and 0.721, respectively. Except for RF with clinical features alone, no significant difference was observed between the CNN model with clinical features and other three models. ROC curves of each model in the training set are shown in Fig. S1.

When two junior radiologists classifying the solid nodules in the testing set, they achieved AUCs of 0.615–0.755, sensitivities of 0.778–0.990, specificities of 0.452–0.519 and accuracies of 0.611–0.749. The results indicated that established models demonstrated higher specificities while the radiologists achieved higher sensitivities. Detailed diagnostic performance of each model and radiologist in the testing set are summarized in Table 2.

**Table 2:** Predictive performance of different classification models and junior radiologists in the testing set

Model or radiologist	Sensitivity	Specificity	Accuracy	AUC
**RF + Clinical**	0.535 [0.437, 0.633]	0.740 [0.656, 0.824]	0.640 [0.574, 0.706]	0.721 [0.651, 0.791]*
**RF + Radiomics**	0.747 [0.661, 0.833]	0.606 [0.512, 0.700]	0.675 [0.611, 0.739]	0.778 [0.738, 0.858]
**RF + Combined**	0.616 [0.520, 0.712]	**0.788 [0.709, 0.867]**	0.704 [0.641, 0.767]	0.811 [0.713, 0.839]
**CNN**	0.758 [0.674, 0.842]	**0.788 [0.709, 0.867]**	0.773 [0.715, 0.831]	0.816 [0.758, 0.875]
**CNN+ Clinical**	0.778 [0.696, 0.860]	**0.788 [0.709, 0.867]**	**0.783 [0.726, 0.840]**	**0.819 [0.760, 0.877]**
**Radiologist 1**	0.778 [0.696, 0.860]	0.452 [0.356, 0.548]	0.611 [0.544, 0.678]	0.615 [0.538,0.692]
**Radiologist 2**	**0.990 [0.970, 1.000]**	0.519 [0.423, 0.615]	0.749 [0.689, 0.808]	0.755 [0.688,0.821]

*Significant difference was found between the CNN model with clinical features and RF with clinical features by Delong test (*p* < 0.05)

*Abbreviations*: *RF* Random forest, *CNN* Convolutional neural network, *AUC* Area under the receiver operating characteristic curves

Figure 2B shows calibration curves. The CNN model with clinical features achieved the smallest Brier score of 0.177. The Brier score of CNN model without clinical features, RF with clinical features, RF with radiomics features and RF with combined features was 0.183, 0.215, 0.195, and 0.184, respectively.

### Survival analysis

**Fig. 3:**
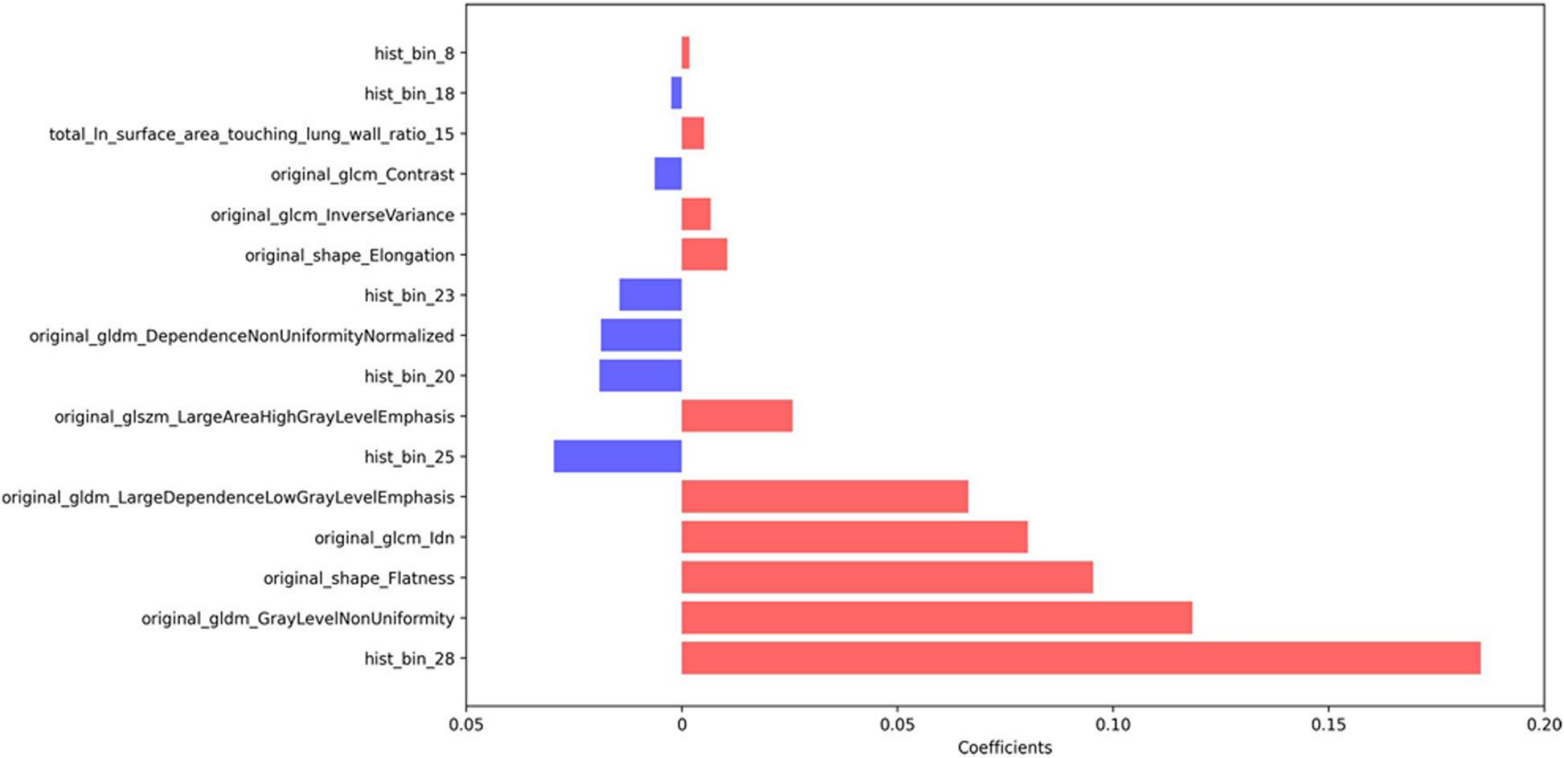
Bar plot of significant radiomics features associated with disease-free survival selected by LASSO analysis

**Fig. 4:**
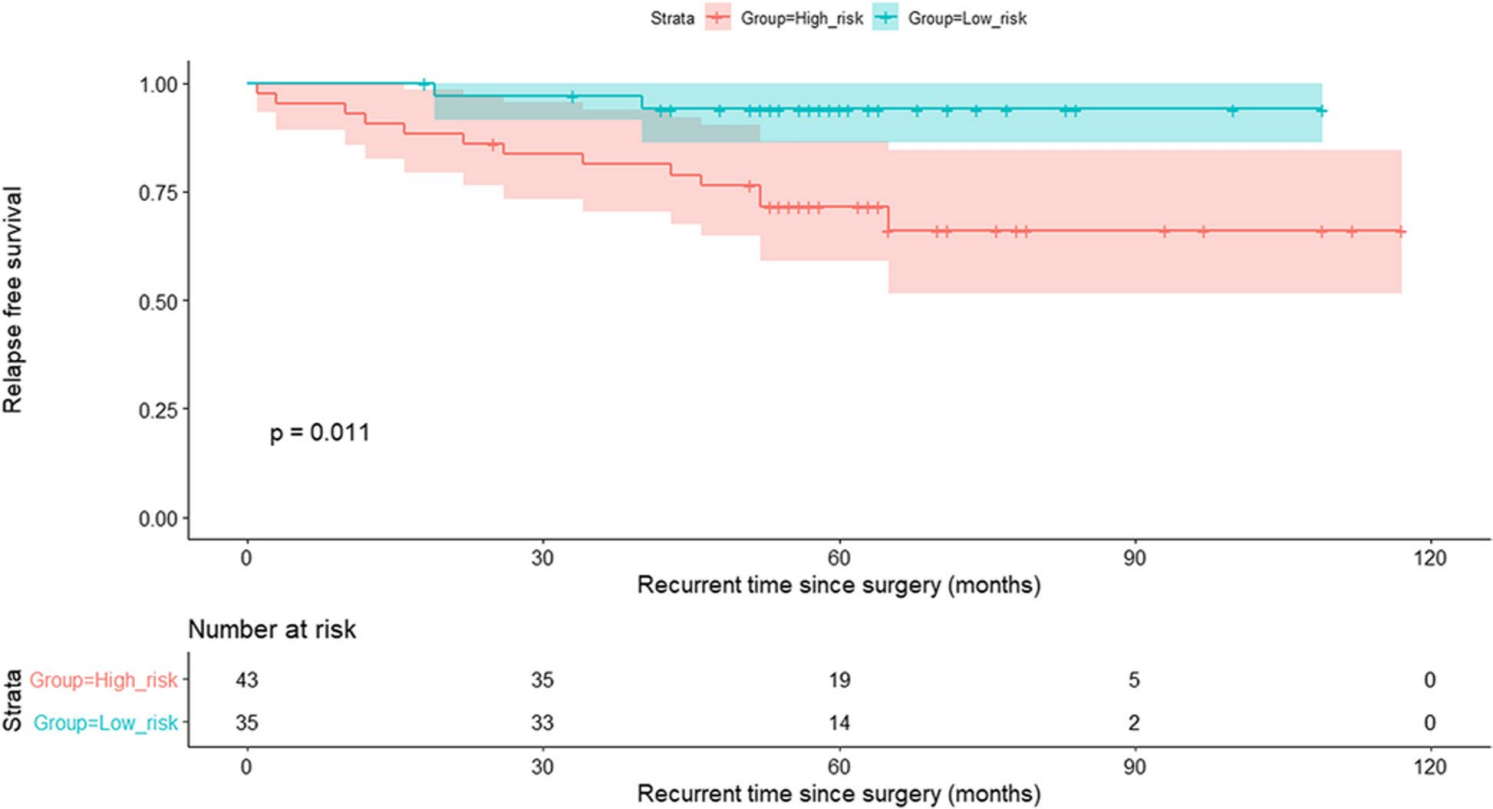
Kaplan-Meier survival curves in the testing set based on radiomics features

From 295 surgically resected adenocarcinomas, survival data were collected. Table 3 summarizes the clinical characteristics of patients in survival analysis. When performing LASSO analysis, sixteen radiomics features were found to be associated with DFS (Fig. 3). All these significant features were used to calculate the Rad-score and according to a cutoff point of 0.183 based on X-tile, the patient was classified into low-risk or high-risk group. The Kaplan-Meier survival analysis showed that DFS between the low-risk and high-risk groups were statistically different. In the testing set, the mean DFS was 104 months (95% CI, 98–110 months) for the low-risk group and 89 months (95% CI, 75–102 months) for the high-risk group (*P* = 0.011, Fig. 4). The Kaplan-Meier survival curve in the training set is shown in Fig. S2.

**Table 3:** Clinical characteristics of patients in survival analysis

Characteristics	Training (*N* = 217)	Testing (*N* = 78)	*P* value
Stage			0.378
IA	132 (60.8)	52 (66.7)	
IB	67 (30.9)	23 (29.5)	
II-III	18 (8.3)	3 (3.8)	
Follow-up time (month)			0.637
Mean ± standard deviation	55 ± 21.8	58 ± 23.4	
Median (25th, 75th)	57 (47–66)	57 (51–66)	
Number of recurrence			
All	47 (21.7)	15 (19.2)	0.652
At 1 year	13 (6.0)	3 (3.8)	
At 2 years	21 (9.7)	7 (9.0)	
At 3 years	29 (13.4)	9 (11.5)	
DFS, mean with 95% CI (month)			
All	90 (84, 95)	99 (90, 107)	0.634
Low-risk group based on Rad-score	96 (92, 101)	104 (98, 110)	–
high-risk group based on Rad-score	71 (60, 81)	89 (75, 102)	–

Unless specified, data in parentheses are percentages

*Abbreviations*: *DFS* Disease-free survival, *CI* Confidence interval

**Fig. 5:**
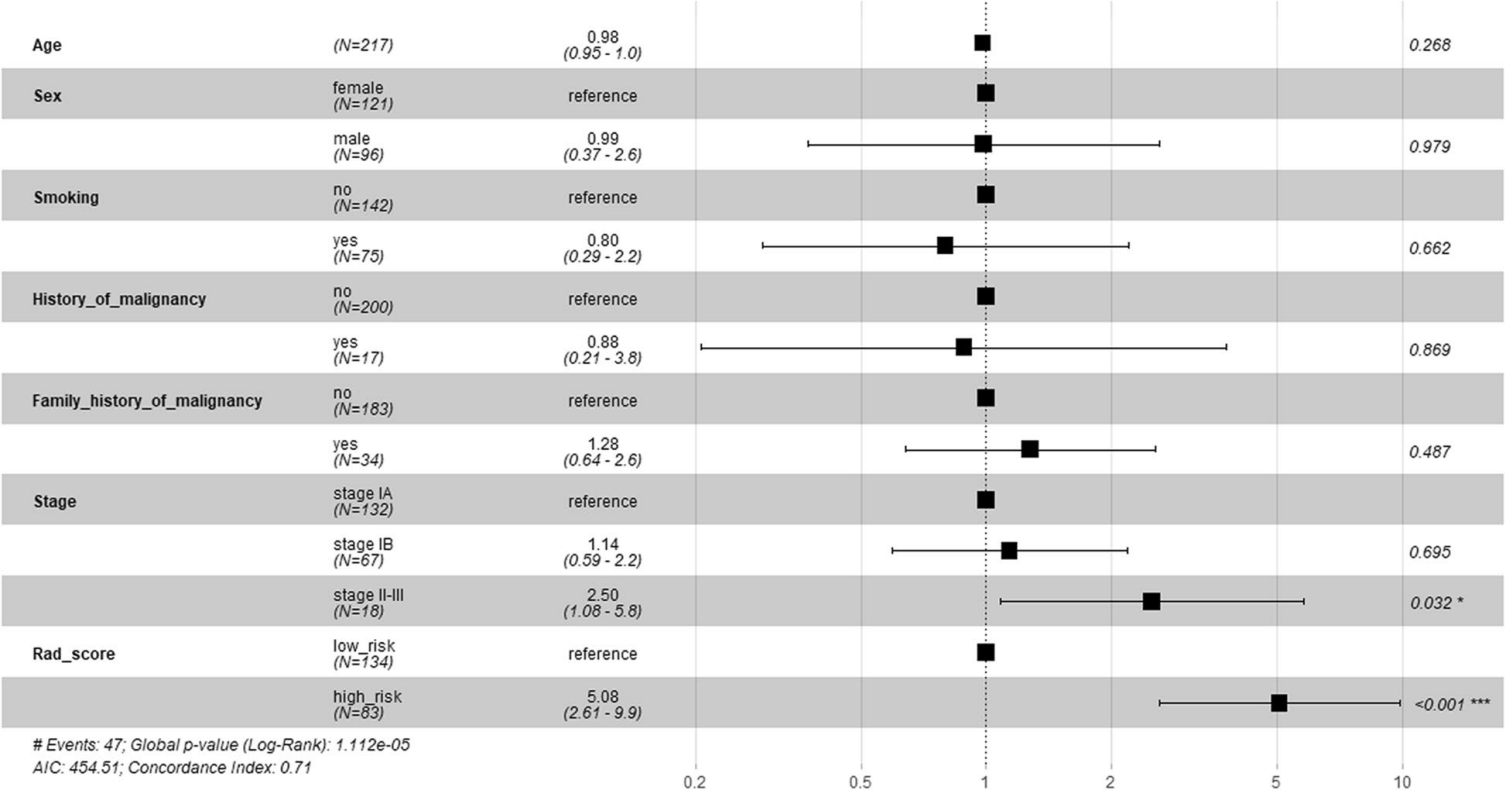
Random Forest of the multivariate Cox regression analysis

In addition, the result of multivariate Cox regression analysis showed that the clinical stage [hazard ratio (HR), 2.50, 95% CI, 1.08–5.80, *P* = 0.032) and Rad-score (HR, 5.08, 95% CI, 2.61–9.90, *P* < 0.001) were two independent predictors of DFS (Fig. 5). The prognostic value of radiomics features was also proved among stage I patients (Fig. S3 and S4). Furthermore, we assessed the effectiveness of malignancy-score derived from the CNN model with clinical features in prognostic analysis, but the results indicated that the malignancy-score derived from CNN model might be not as effective as the score derived from radiomics features (Fig. S5).

## Discussion

The current study evaluated the diagnostic and prognostic value of radiomics and deep learning technologies for patients with solid pulmonary nodules. We found the established CNN models demonstrated the best diagnostic performance, followed by radiomics models and then the model developed from clinical variables alone. The established CNN models and radiomics models performed slightly better than the two junior radiologists. Besides, it was found that the Rad-score based on sixteen radiomics features were important in predicting DFS of patients with solid adenocarcinomas.

Radiomics and deep learning technologies have been playing an important role in cancer research [11, 12]. Similarly, studies predicting risk of solid nodules of 6 mm to 15 mm found the CNN and radiomics model demonstrated an AUC of 0.93 and 0.97, respectively [14, 17]. Wu et al. studied solid nodules smaller than 20 mm and found the radiomics model achieved an AUC of 0.89 [15]. Besides, Yang et al. investigated solid lung adenosarcomas and granulomas and the AUCs of combined radiomics and clinical risk factors were 0.82–0.84 [16]. Feng et al. established a deep learning nomogram to differentiate tuberculosis granulomas from lung adenocarcinomas, which yielded AUCs of 0.89–0.81 [19]. The current study investigated solid pulmonary nodules less than 30 mm in a larger sample size, and found the AUCs of CNN models and radiomics models were 0.78–0.82. Hence, it’s advisable to apply radiomics and deep learning technologies in solid nodule management in future.

In the radiomics models, most selected predictors were texture and histogram distribution features. The texture features can measure the spatial inter-dependency or co-occurrence of information across adjacent voxels [26]. Specifically, the GLCM feature accounted for the largest proportion of selected texture features in the current study, which measures the value of texture images with pixels of the same gray level and is mainly applied for linear texture analysis. In previous studies, GLCM is one of the most commonly used radiomics features, which may be associated with the spatial heterogeneity of lung lesions [27, 28]. The histogram distribution features represent the distribution of gray pixels in the intensity image, which may characterize different subtypes of nodules with varying degrees of density properties [29]. However, when compared with similar studies on lung cancer risk prediction, the selected radiomics features for modeling were not exactly the same, which may be due to different strategies applied in radiomics feature extraction and the heterogeneity of the dataset [14–16].

We also evaluated the prognostic value of radiomics and deep learning technologies for patients with solid adenocarcinomas. It was reported that the recurrence rate of early-stage non-small cell lung cancer is still substantial about 15–38.5% [30]. For adenocarcinomas, the recurrence rate is significantly high in micropapillary-predominant and solid-predominant subtypes [31]. In the current study, totally 62 adenocarcinomas (21%) relapsed and the median DFS was 26 months. Hence, it’s essential to identify those who will suffer from disease relapse. Our results indicated that the Rad-score based on sixteen radiomics features was an independent predictor of DFS, with an even higher HR value than clinical stage. Similar findings were reported in previous studies [32–34]. Xie et al. found age, pathologic TNM stage, histologic subtype and the radiomics signature were predictors of DFS in lung adenocarcinomas [34]. In addition, Huang et al. also found the radiomics signatures were significantly associated with DFS of non-small cell lung cancer, and the radiomics-based nomogram resulted in better performance than that with the clinical-pathologic variables [35]. However, it seemed that the CNN signatures were not as predictive as radiomics signatures from our current data, which could be caused by the small sample size in prognostic analysis.

There were some limitations need to be considered when interpreting our results. Firstly, this was a single center study and models weren’t externally validated. Previous studies have shown that when revalidated with external data, the performance of models may be reduced due to heterogeneous acquisition protocols and patient populations [36, 37]. Secondly, the current study was retrospectively carried out. Therefore, the CT images used for radiomics and deep learning analysis were not obtained from the same scanner, which may reduce the stability of risk models.

## Conclusions

On the one hand, it was found that the CNN models and radiomics models demonstrated good performance in predicting the malignancy of solid nodules, superior to the model based on clinical variables alone. On the other hand, radiomics features demonstrated potential to predict the DFS of patients with solid adenocarcinomas.

## Supplementary Information

**Additional file 1: Table S1.** Distribution of the CT manufacturer in the training and testing sets. **Table S2.** Distribution of the CT slice thickness in the training and testing sets. **Fig. S1.** ROC curves of different classification models in the training set. RF: random forest, CNN: convolutional neural network. **Fig. S2.** Kaplan-Meier survival curves in the training set based on radiomics features. **Fig. S3.** Kaplan-Meier survival analysis among stage I patients (testing set). The disease-free survival between the low-risk and high-risk groups were statistically different (*P* < 0.05). **Fig. S4.** Random forest of the multivariate Cox regression analysis among stage I patients. Rad-score (HR, 4.92, 95% CI, 2.44–9.90, *P* < 0.001) was the only independent predictor of disease-free survival. **Fig. S5.** Kaplan-Meier survival curves with malignancy-score derived from the CNN model with clinical features in the training (A) and testing (B) set.

## Data Availability

Please contact author (Bojiang Chen) for data requests.

## Abbreviations

SSN: Subsolid nodule
CNN: Convolutional neural network
RF: Random forest
LASSO: Least absolute shrinkage and selection operator
ROC: Receiver operating characteristic curve
AUC: Area under the ROC curve
DFS: Disease-free survival
CI: Confidence interval
