# Supplementary material for: The diagnostic and prognostic value of radiomics and deep learning technologies for patients with solid pulmonary nodules in chest CT images
Source: BMC Cancer. 2022 Nov 1;22:1118. doi: 10.1186/s12885-022-10224-z (PMC9628173; doi:10.1186/s12885-022-10224-z)
Supplement: Supplementary file 1 — Additional file 1: Table S1. Distribution of the CT manufacturer in the training and testing sets. Table S2. Distribution of the CT slice thickness in the training and testing sets. Fig. S1. ROC curves of different classification models in the training set. RF: random forest, CNN: convolutional neural network. Fig. S2. Kaplan-Meier survival curves in the training set based on radiomics features. Fig. S3. Kaplan-Meier survival analysis among stage I patients (testing set). The disease-free survival between the low-risk and high-risk groups were statistically different (P < 0.05). Fig. S4. Random forest of the multivariate Cox regression analysis among stage I patients. Rad-score (HR, 4.92, 95% CI, 2.44–9.90, P < 0.001) was the only independent predictor of disease-free survival. Fig. S5. Kaplan-Meier survival curves with malignancy-score derived from the CNN model with clinical features in the training (A) and testing (B) set. [file 12885_2022_10224_MOESM1_ESM.docx]

**Table S1** Distribution of the CT manufacturer in the training and testing sets.

| Manufacturer | Training (N=517) | Testing (N=203) | Total |
| --- | --- | --- | --- |
| Siemens | **471** | **188** | **659** |
| Sensation 16 | 11 (2.3) | 10 (5.3) | 21 (3.2) |
| Somatom definition flash | 460 (97.7) | 178 (94.7) | 638 (96.8) |
| Philips | **30** | **12** | **42** |
| Brilliance 16 | 9 (30.0) | 5 (41.7) | 14 (33.3) |
| Brilliance 64 | 21 (70.0) | 7 (58.3) | 28 (66.7) |
| GE medical systems | **16** | **3** | **19** |
| Revolution CT | 16 (100.0) | 3 (100.0) | 19 (100.0) |

Note: Data is frequency or frequency (proportion). No significant difference was found between the training set and testing set on the CT manufacturer (p>0.05, Chi-square test).

**Table S2** Distribution of the CT slice thickness in the training and testing sets.

| Slice thickness | Training (N=517) | Testing (N=203) | Total |
| --- | --- | --- | --- |
| 1 mm | 488 (94.4) | 194 (95.6) | 682 (94.7) |
| Others | 29 (5.6) | 9 (4.4) | 38 (5.3) |

Note: Data is frequency (proportion). No significant difference was found between the training set and testing set on the slice thickness (p>0.05, Chi-square test).

- **The predictive performance of diagnostic models in the training set**


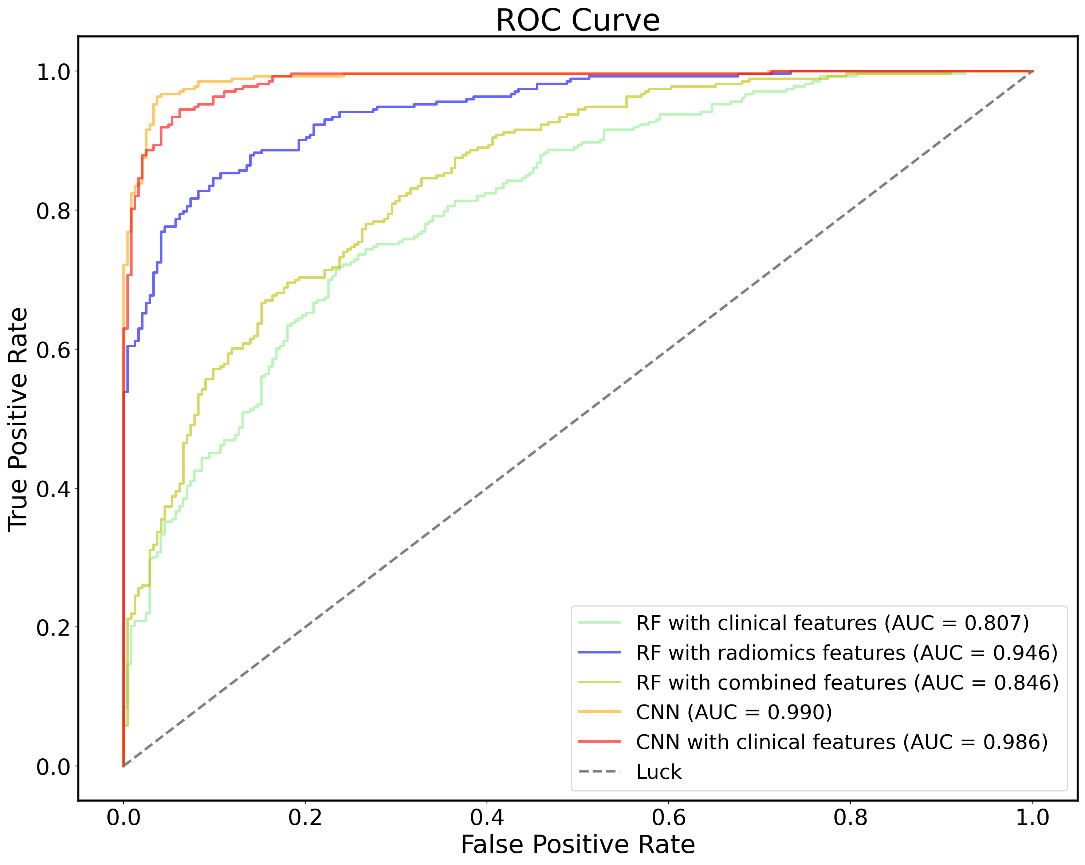


**Fig. S1.** ROC curves of different classification models in the training set. RF: random forest, CNN: convolutional neural network.

- **The Kaplan-Meier survival curves in the training set.**


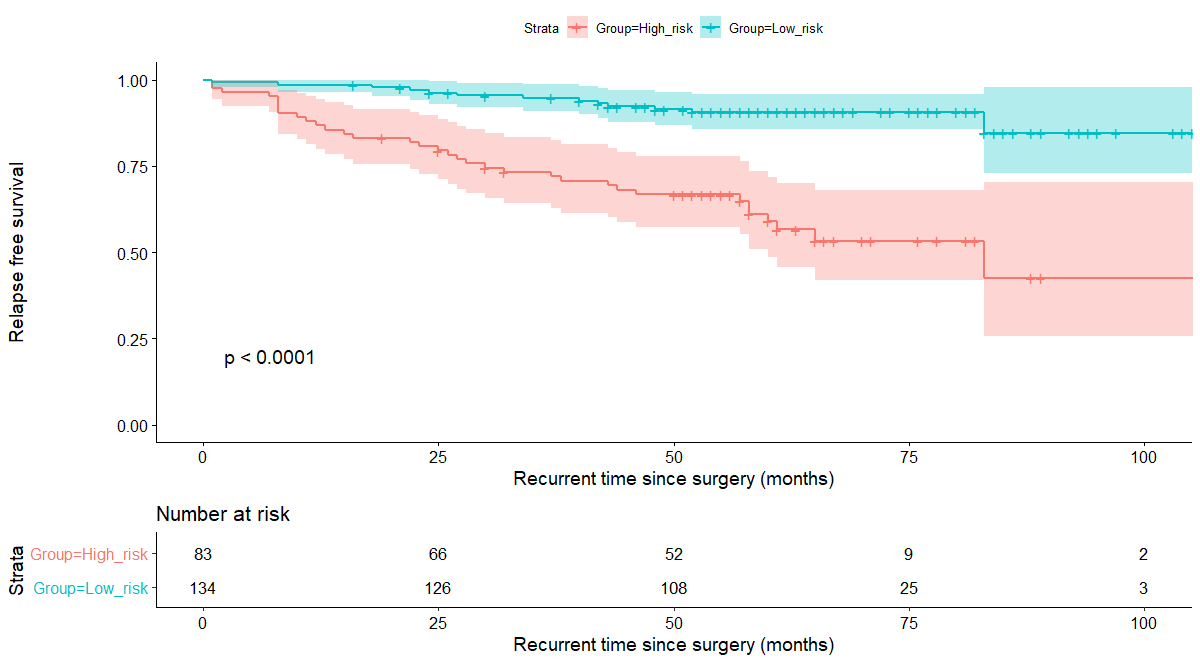


**Fig. S2.** Kaplan-Meier survival curves in the training set based on radiomics features.

- **Survival analysis among stage I patients with solid adenocarcinomas**


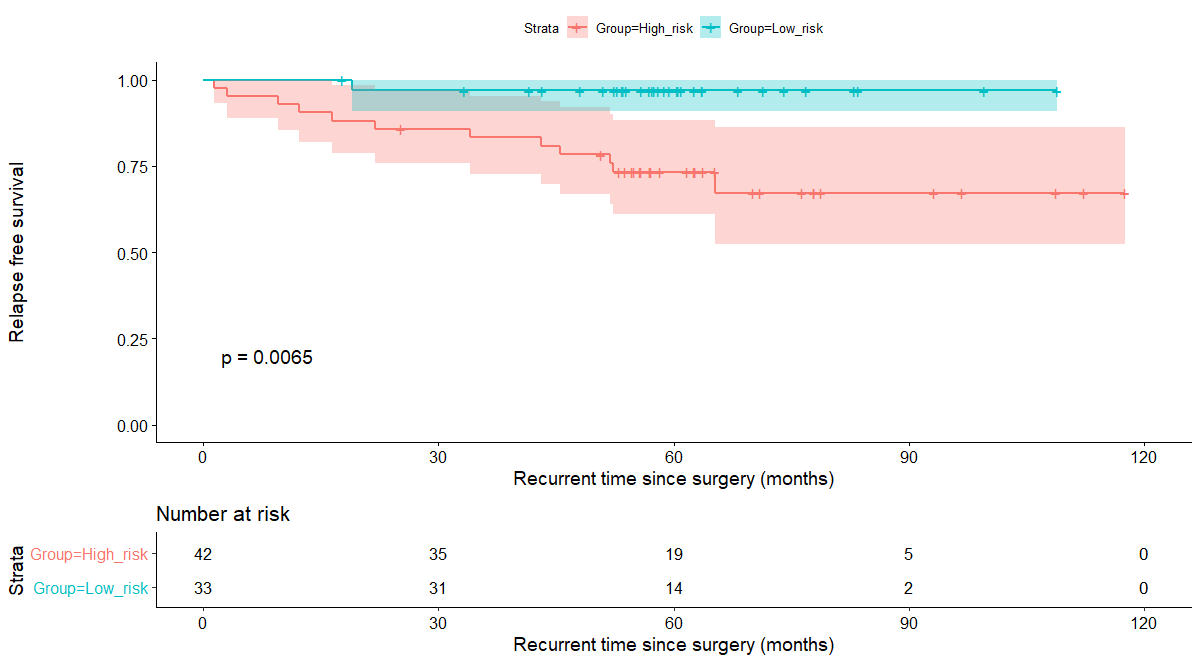


**Fig. S3.** Kaplan-Meier survival analysis among stage I patients (testing set). The disease-free survival between the low-risk and high-risk groups were statistically different (P<0.05)


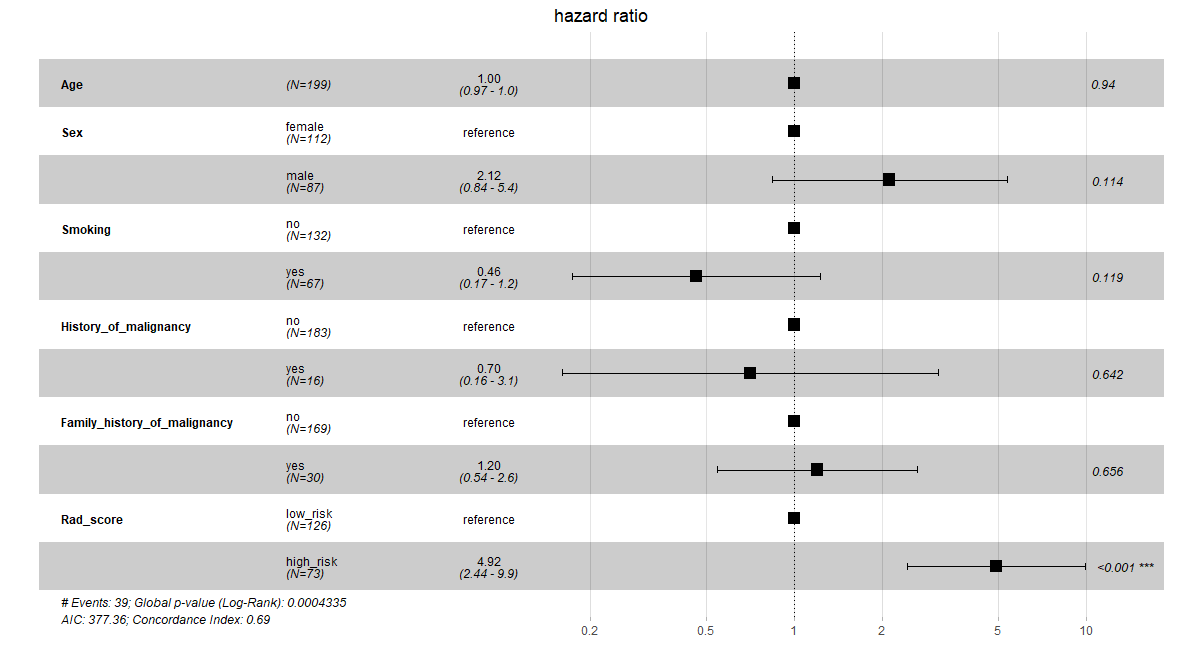


**Fig. S4.** Random forest of the multivariate Cox regression analysis among stage I patients. Rad-score (HR, 4.92, 95% CI, 2.44-9.90, P<0.001) was the only independent predictor of disease-free survival.

- **Survival analysis based on the CNN model**

Furthermore, we assessed the effectiveness of malignancy-score derived from the CNN model with clinical features in prognostic analysis. However, as shown in **Figure S5**, no significant difference was found between the low-risk and high-risk groups by the Kaplan-Meier survival analysis (P>0.05), which indicated that the malignancy-score derived from CNN model might be not as effective as the score derived from radiomics features.


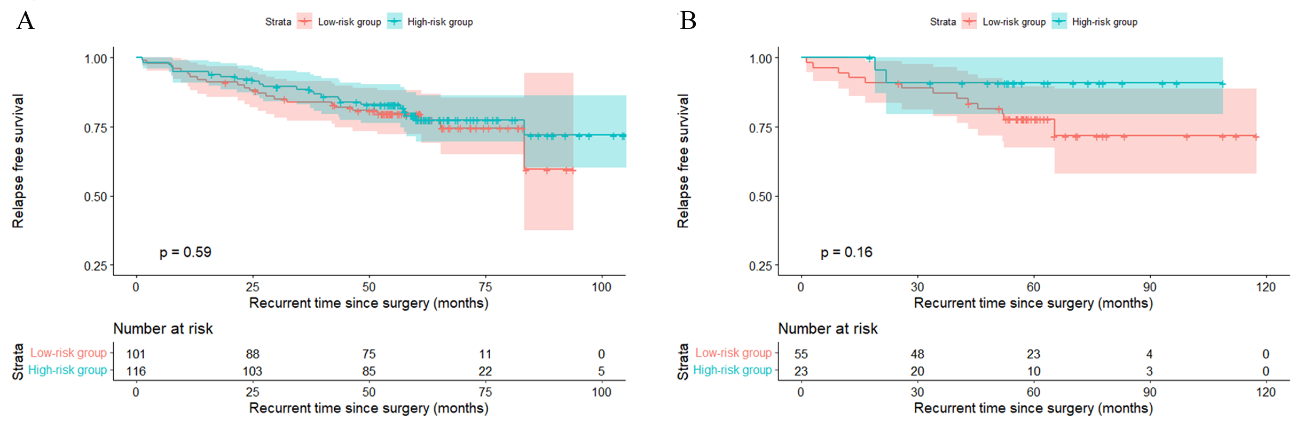


**Fig. S5.** Kaplan-Meier survival curves with malignancy-score derived from the CNN model with clinical features in the training (A) and testing (B) set.
